# Colorectal Cancer Incidence, Inequalities, and Prevention Priorities in Urban Texas: Surveillance Study With the “surveil” Software Package

**DOI:** 10.2196/34589

**Published:** 2022-08-16

**Authors:** Connor Donegan, Amy E Hughes, Simon J Craddock Lee

**Affiliations:** 1 Department of Population and Data Sciences University of Texas Southwestern Medical Center Dallas, TX United States; 2 Department of Geospatial Information Sciences The University of Texas at Dallas Dallas, TX United States; 3 Department of Population Health University of Kansas Medical Center Kansas City, KS United States

**Keywords:** Bayesian analysis, cancer prevention, colorectal cancer, health equity, open source software, public health monitoring, time-series analysis

## Abstract

**Background:**

Monitoring disease incidence rates over time with population surveillance data is fundamental to public health research and practice. Bayesian disease monitoring methods provide advantages over conventional methods including greater flexibility in model specification and the ability to conduct formal inference on model-derived quantities of interest. However, software platforms for Bayesian inference are often inaccessible to nonspecialists.

**Objective:**

To increase the accessibility of Bayesian methods among health surveillance researchers, we introduce a Bayesian methodology and open source software package, surveil, for time-series modeling of disease incidence and mortality. Given case count and population-at-risk data, the software enables health researchers to draw inferences about underlying risk and derivative quantities including age-standardized rates, annual and cumulative percent change, and measures of inequality.

**Methods:**

We specify a Poisson likelihood for case counts and model trends in log-risk using the first-difference (random-walk) prior. Models in the surveil R package were built using the Stan modeling language. We demonstrate the methodology and software by analyzing age-standardized colorectal cancer (CRC) incidence rates by race and ethnicity for non-Latino Black (Black), non-Latino White (White), and Hispanic/Latino (of any race) adults aged 50-79 years in Texas’s 4 largest metropolitan statistical areas between 1999 and 2018.

**Results:**

Our analysis revealed a cumulative decline of 31% (95% CI –37% to –25%) in CRC risk among Black adults, 17% (95% CI –23% to –11%) for Latino adults, and 35% (95% CI –38% to –31%) for White adults from 1999 to 2018. None of the 3 observed groups experienced significant incidence reduction in the final 4 years of the study (2015-2018). The Black-White rate difference (per 100,000) was 44 (95% CI 30-57) in 1999 and 35 (95% CI 28-43) in 2018. Cumulatively, the Black-White gap accounts for 3983 CRC cases (95% CI 3746-4219) or 31% (95% CI 29%-32%) of total CRC incidence among Black adults in this period.

**Conclusions:**

Stalled progress on CRC prevention and excess CRC risk among Black residents warrant special attention as cancer prevention and control priorities in urban Texas. Our methodology and software can help the public and health agencies monitor health inequalities and evaluate progress toward disease prevention goals. Advantages of the methodology over current common practice include the following: (1) the absence of piecewise linearity constraints on the model space, and (2) formal inference can be undertaken on any model-derived quantities of interest using Bayesian methods.

## Introduction

Monitoring disease incidence rates is fundamental to public health research and practice. Vital statistics systems, cancer registries, and other disease-specific monitoring programs provide critical data resources for public health research, and valid interpretation of these data requires formal modeling.

Joinpoint regression modeling (JRM) is a commonly employed, National Cancer Institute–endorsed method for monitoring incidence and mortality rates [1-4]. JRM fits a piecewise linear time trend to (log-) incidence rates. Nonetheless, piecewise linearity conflicts with subject matter expertise insofar as we “do not really believe that cancer rates change abruptly” [1] and some trends are “obviously nonlinear” [3]. Further, standard JRM methodology systematically underreports the uncertainty of estimates because the SEs are conditional on an iterative model selection procedure [5].

We present a Bayesian methodology and open source software package for routine disease surveillance. The models are appropriate for time-series count data aggregated across evenly spaced time periods. The models assign the Poisson likelihood to observed counts conditional on unknown risk; time trends in risk are modeled by assigning the first-difference (random-walk) prior distribution to the log-rates. Binomial models for nonrare events are also implemented. Strengths of the method include its parsimony, the absence of linearity constraints, and the use of Bayesian inference [6-12] to summarize knowledge of disease risk as well as model-derived quantities of interest, such as age-standardized rates and measures of inequality [9,11,12]. The methodology is freely available through the surveil R software package [13,14].

We demonstrate use of the surveil R package by analyzing urban colorectal cancer (CRC) incidence in Texas. “Eliminating cancer disparities” is purportedly a “cross-cutting aim” of the Cancer Prevention and Research Institute of Texas’s (CPRIT’s) *2018 Texas Cancer Plan,* but the plan conspicuously lacks disparity-related goals and metrics [15]. Racial-ethnic inequalities in CRC are of long-standing concern, including the Black-White incidence and mortality differences that emerged in the early 1990s [16,17]. We examine CRC incidence by race and ethnicity in the 4 largest metropolitan areas in Texas, using our Poisson time-series models. We also examine CRC incidence inequalities and their change over time [18]. We conclude with comments on CRC prevention priorities for Texas, lessons from successful CRC screening efforts, and limitations of this analysis.

## Methods

### Model Specification

The surveil R package implements Poisson random-walk models. For time period *t=*{*1,…,n*}, we assign the Poisson probability distribution to the observed case counts, y_t,_ conditional on a given level of risk, exp(η_t_), and population at risk, P_t_:

y_t_~Pois(P_t_ * exp(η_t_))

Alternatively, the binomial likelihood may be used:

y_t_~Binom(P_t,_ g^-1^(η_t_)),

where g^-1^(x)=exp(x)/(1+exp(x)) is the inverse-logit function.

We assign the first-difference (random-walk) model to the log- or logit-transformed risk parameters, consistent with our knowledge that disease risk tends to vary smoothly over time:

η_t_~Gau(η_t-1_, τ^2^), t>1

This and related intrinsic Gaussian Markov random field specifications are extensively studied models for time trend analyses [19,20].

By default, surveil prior distributions are diffuse for most applications, and users can adjust them to match their subject matter knowledge. The log- or logit-transformed risk for *t=1* (η_1_), requires a prior distribution (because *t=0* does not exist). For a rare disease, the following prior is diffuse:

η_1_~Gau(-5, 5^2^)

It is centered on a rate of e^-5^=673 per 100,000 and spreads the prior probability across a wide range of values. Changes in log-rates are small, such that surveil’s following default prior is also diffuse:

τ~Gau^+^(0, 1^2^)

This base model specification may be extended for multiple correlated time series, such as observations of multiple demographic groups. If **η_t_** is the vector of log-rates for *k* groups at time *t*, then

**η_t_**~Gau(**η_t-1_**, ∑)

introduces a covariance structure through the multivariate normal distribution [21]. We decompose the *k-*by-*k* covariance matrix, ∑, into a vector of scale parameters and a correlation matrix, and use the LKJ distribution as a prior for the correlation matrix [22].

### Bayesian Inference

The models were built in Stan, a state-of-the-art platform for Bayesian inference with Markov chain Monte Carlo (MCMC) [22,23]. Stan uses a Hamiltonian Monte Carlo algorithm to draw samples from user-specified joint probability distributions [9,11,24]. Model results are summarized by surveil, which reports estimates (means of marginal posterior distributions) with 95% CIs (Textbox 1).

**The surveil R package.**

The surveil R package is freely available and archived on the Comprehensive R Archive Network. Basic use of the software requires only introductory-level R programming skills. Tables downloaded from the CDC Wonder database are automatically in the expected format. The model-fitting function, stan_rw, returns a summary of results (estimates with 95% CIs) and Markov chain Monte Carlo (MCMC) samples.

The package supports a streamlined workflow for analyzing disease incidence data. It produces publication-quality visualizations using ggplot2 [25] and enables researchers to make health equity an integral component of surveillance research. The models were built in the Stan modeling language, a robust, stable, state-of-the-art platform for Bayesian inference, providing built-in MCMC diagnostics and visualization methods.

Using MCMC, probability statements can be made about any quantity of interest that is derived from model parameters [9,11,12]. Functions in the surveil package return probability distributions for model-derived quantities such as annual and cumulative percent change, age-standardized rates, the Theil inequality index [26], and a variety of pairwise inequality measures (Textbox 2). The Theil index measures discrepancies between each group’s share of disease burden and their share of the population; owing to its fractal structure, it can be used to measure inequality across geographically nested populations [27-29].

When working with age-standardized rates, excess cases (ECs) must be calculated separately for each age stratum and then summed across age groups (Textbox 3). Dividing the resulting ECs by total risk provides an age-standardized measure of proportion attributable risk (PAR). For age-standardized rates, this method of calculating the PAR may be preferred over the rate ratio (RR) as a measure of relative inequality because the PAR better reflects the actual age distribution. If an RR is still preferred and the PAR is less than 1, the PAR can be converted to its equivalent RR using RR=(1/PAR)/(1/PAR-1).

**Measures of pairwise inequality provided by surveil.**

Rate ratio (RR)=R_d_/R_a_, where R is the incidence rate, and subscripts “a” and “d” represent the advantaged and disadvantaged demographic groups, respectively.

- Rate difference (RD)=R_d_–R_a_
- Proportion attributable risk (PAR)=RD/R_d_
- Excess cases (EC)=RD×P_d_, where P represents the populations at risk.
- Cumulative EC=Σ_t_ EC_t_, where the subscript “t” represents the time period.
- Cumulative PAR=Σ_t_ EC_t_/Σ_t_ (R_dt_×P_dt_)

**Age-standardized measures of pairwise inequality provided by surveil.**

Rate ratio (RR)=SR_d_/SR_a_, where “SR” is the age-standardized incidence rate, and subscripts “a” and “d” represent the advantaged and disadvantaged demographic groups, respectively.

- Rate difference=SR_d_–SR_a_
- Excess cases (EC)=Σ_i_ (R_di_–R_ai_)×P_di_, where “P” represents the populations at risk, and subscript “i” represents the age groups.
- Proportion attributable risk (PAR)=EC/Σ_i_R_di_×P_di_
- Cumulative EC=Σ_t_ EC_t_, where “t” represents the time periods.
- Cumulative PAR=Σ_t_ EC_t_/Σ_t_ Σ_i_ (R_dit_×P_dit_)

### CRC Incidence in Urban Texas

We gathered publicly available age-specific (50-79 years) data on CRC incidence and population at risk, between 1999 and 2018, by race and ethnicity in the 4 largest metropolitan statistical areas (MSAs) in Texas (centered in Austin, Dallas, Houston, and San Antonio). Uncensored data for this age range are publicly available at the level of 5-year age groups for Hispanic/Latino (all racial groups combined), non-Latino Black or African American (Black), and non-Latino White (White) populations. CRC data for Asian Pacific Islanders are not available for 5-year age groups but are available for the aggregate 50-79–year-old population. Data for American Indians/Alaska Natives are not available [30].

We modeled CRC incidence by race-ethnicity and 5-year age group for the 4 MSAs combined using surveil’s Poisson first-difference models. We calculated age-standardized rates using direct age-standardization and the 2000 US standard million population [12]. While remaining cognizant of data limitations, we also modeled age-specific (50-79 years) CRC incidence with Poisson first-difference models in order to examine CRC risk among Asian Pacific Islander residents.

We examined rates of change by calculating the average annual percent change (AAPC) per 4-year period. The sole purpose of aggregating to 4-year periods is to stabilize the estimates. We measure Black-White inequality by the rate difference (RD), PAR, and ECs. Probability distributions for all quantities of interest were obtained using MCMC analysis. For each model, we drew 6000 samples from each of 4 MCMC chains, discarding the first 3000 samples of each chain as warm-up. Before analyzing the results, we confirm that MCMC samples converge on a single distribution using the split R-hat statistic and that MCMC SEs are sufficiently small [11]. We summarize posterior distributions using the mean and 95% CI.

## Results

### Aggregate MSA Trends

**Figure 1:**
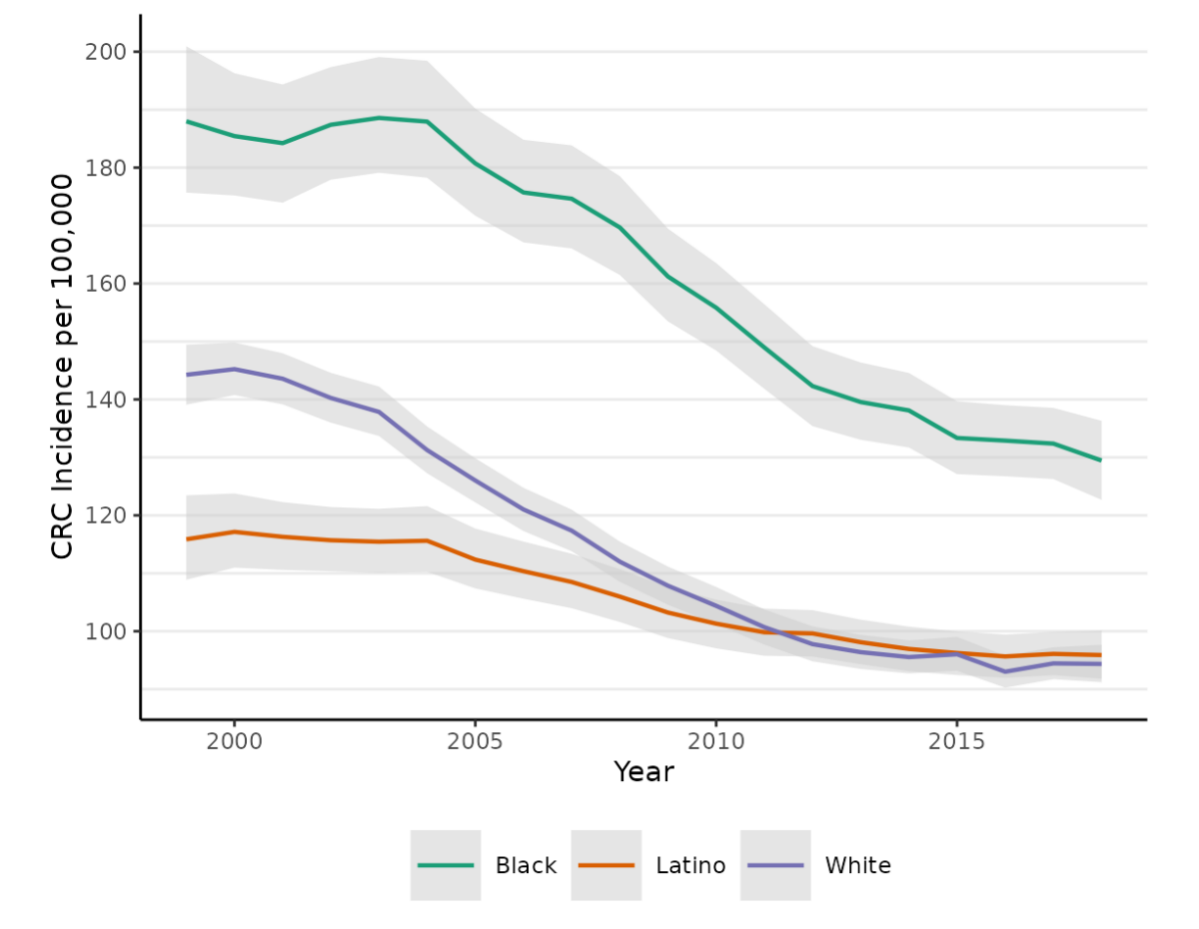
Age-standardized incidence rates of colorectal cancer (CRC) per 100,000 by race-ethnicity among adults aged 50-79 years between 1999 and 2018 in 4 Texas metropolitan statistical areas.

CRC incidence declined substantially between 1999 and 2018 (Figure 1 and Table 1). Age-standardized CRC risk declined 31% (95% CI –37% to –25%) for Black adults from a rate per 100,000 of 188 (95% CI 176-201) in 1999 to 129 (95% CI 123-136) in 2018. CRC risk among White adults decreased by 35% (95% CI –38% to –31%), from 144 per 100,000 (95% CI 140-150) in 1999 to 94 (95% CI 91-98) in 2018. Among Latino adults, CRC risk decreased by 17% (95% CI –23% to –11%), from 116 (95% CI 109-123) in 1999 to 96 (95% CI 92-100) in 2018. Results from the age-specific models (Table 2), while subject to some amount of confounding by age, indicate that CRC risk was lower for Asian Pacific Islanders than for the other groups examined and that Asian Pacific Islanders experienced the smallest cumulative change in risk (if any), which was –11% (95% CI –25% to 3%).

**Table 1:** Levels and cumulative percent change of age-standardized risk of colorectal cancer (CRC) per 100,000 among adults aged 50-79 years, in Texas’s 4 largest metropolitan statistical areas between 1999 and 2018.

	Age-standardized CRC risk in 1999, risk (95% CI)	Age-standardized CRC risk in 2018, risk (95% CI)	Percent (%) change (95% CI)
Black	188 (176 to 201)	129 (123 to 136)	–31 (–37 to –25)
Latino	116 (109 to 123)	96 (92 to 100)	–17 (–23 to –11)
White	144 (140 to 150)	94 (91 to 98)	–35 (–38 to –31)

**Table 2:** Levels and cumulative percent change of age-specific risk of colorectal cancer (CRC) per 100,000 among adults aged 50-79 years (not age-standardized), in Texas’s 4 largest metropolitan statistical areas between 1999 and 2018.

	Non–age-standardized CRC risk in 1999, risk (95% CI)	Non–age-standardized CRC risk in 2018, risk (95% CI)	Percent (%) change (95% CI)
Asian Pacific Islander	75 (66 to 88)	67 (61 to 73)	–11 (–25 to 3)
Black	170 (160 to 182)	122 (115 to 128)	–28 (–34 to –22)
Latino	103 (97 to 109)	86 (83 to 90)	–16 (–22 to –9)
White	135 (130 to 140)	95 (91 to 98)	–30 (–34 to –26)

**Figure 2:**
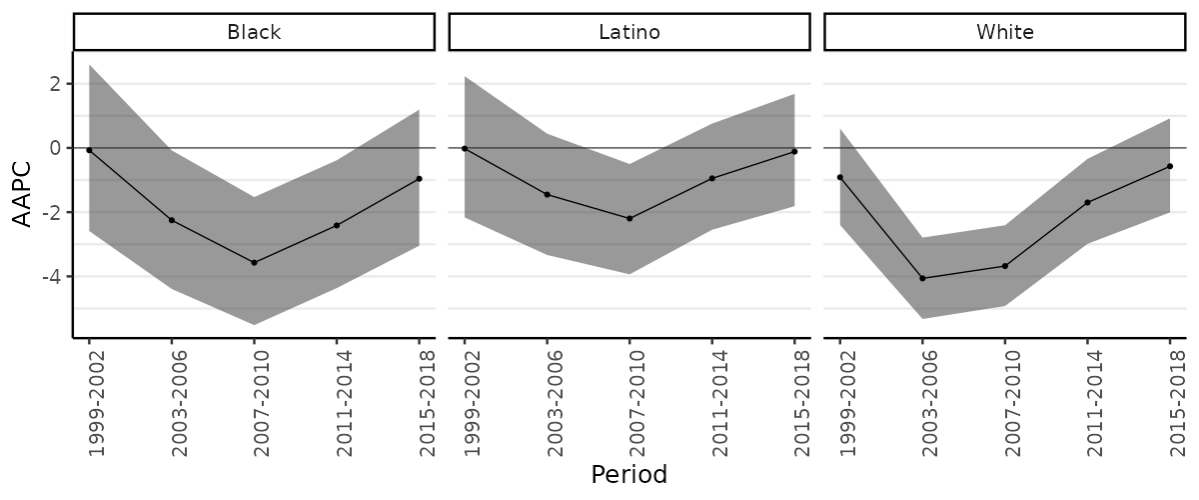
Average annual percent change (AAPC) in age-standardized incidence rates of colorectal cancer (CRC) by 4-year period between 1999 and 2018.

AAPC by 4-year period shows that the most rapid progress on CRC prevention was achieved (roughly) between 2003 and 2014, and that progress appears to have stalled since then (Figure 2). For example, from 2007 to 2010, AAPC for Black, Latino, and White residents, respectively, was –3.7 (95% CI –5.5 to –1.5), –2.2 (95% CI –3.9 to –0.5), and –3.7 (95% CI –4.9 to –2.4). Of these 3 groups, none experienced any robust reduction in CRC risk over the most recent period (2015-2018).

**Figure 3:**
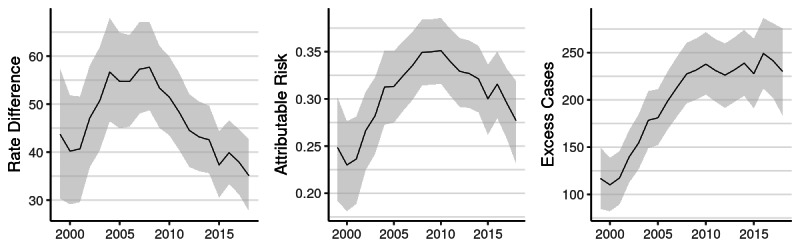
Black-White inequality in the incidence rates of colorectal cancer between 1999 and 2018: rate difference per 100,000, proportion attributable risk, and excess cases.

By multiple measures, aggregate Black-White inequality increased between 1999 and 2008 and then decreased or stabilized by 2018 (Figure 3). The RD increased from 44 per 100,000 (95% CI 30-57) in 1999 to 58 (95% CI 49-67) by 2008 and then decreased to 35 (95% CI 28-43) by 2018. Expressed in relative terms as a percentage of total risk among Black adults (PAR), the Black-White gap increased from 25% (95% CI 19%-30%) in 1999 to 35% (95% CI 31%-38%) in 2008 and then decreased to 28% (95% CI 23%-32%) by 2018. Cumulatively, the Black-White gap accounts for 3983 CRC cases (95% CI 3746-4219) or 31% (95% CI 29%-32%) of CRC incidence among Black residents aged 50-79 years. The EC count is a function of both the RD and size of the population at risk; owing to a combination of Black population growth and the persistence of the Black-White gap, the annual number of excess cases increased from 117 (95% CI 85-150) in 1999 to 230 (95% CI 183-276) in 2018. These represent the number of cases that would have been avoided had the level of risk for Black residents equaled that of White residents each year.

## Discussion

### Methodological Contributions

Monitoring disease incidence is a crucial public health task. The ubiquitous JRM method has notable shortcomings, including linearity constraints and overconfident SEs. This paper presents a parsimonious methodology grounded in Bayesian time-series analysis and accessible through the surveil R package. The package also returns probability distributions for annual and cumulative percent change, measures of pairwise inequality, and the Theil inequality index. Using standard MCMC analysis techniques, users may also conduct inference on any user-defined quantity of interest that is a function of model parameters, such as the AAPC. This project aims to make Bayesian analysis accessible to a wider range of researchers while making robust analyses of health inequality integral to surveillance research. The Poisson models discussed here are appropriate for “rare” events (generally, rates of <0.04). Binomial models for nonrare events are also implemented in surveil. The models are designed for the analysis of data from high-quality surveillance or vital statistics systems that have been aggregated across evenly spaced time periods.

### CRC Prevention Priorities

Between 1999 and 2013, robust CRC risk reduction occurred for White and Black residents, the highest-risk racial-ethnic groups for which data are publicly available, while more modest progress was achieved for Latino and Asian Pacific Islander populations. Excess CRC risk among Black adults is the most burdensome and urgent health inequality identified in this analysis. Black-White inequality increased in relative terms before falling toward its previous level, while annual excess cases increased by approximately 190%. From 2015 to 2018, none of the observed groups experienced any substantial progress in terms of CRC risk reduction.

CRC screening by colonoscopy can prevent CRC through the removal of precancerous polyps [31]. Organized CRC screening programs implemented by, respectively, the state of Delaware and Kaiser Permanente Northern California were followed by substantial reductions in CRC incidence and the practical elimination of Black-White differences in CRC incidence rates [32,33]. New York’s Citywide Colon Cancer Control Coalition (C5) provides a third example of an effective and equitable CRC screening program. The C5 effort included, among other things, a public advertising campaign to promote colonoscopy, a patient navigation system, and a voluntary colonoscopy quality improvement initiative with 230 participating gastroenterologists [34].

Given claims that racial segregation is a driver of Black-White cancer inequalities [35-37], it would be insightful and useful to learn how much of the Black-White gap in metropolitan Texas is accounted for by segregated and high-poverty areas. Ongoing research aims to address important limitations of this analysis using the geostan R package—surveil’s spatially oriented companion for public health research [38,39].

### Limitations

Major limitations of this analysis include the absence of data by social class or income, aggregation of data across distinct MSAs, exclusion of the El Paso metropolitan area, and exclusive focus on the highest-risk age groups.

### Conclusions

Public accountability for public health goals requires routine monitoring of health outcomes and inequalities. surveil can help health agencies and the public in defining goals and monitoring outcomes. Our analysis of CRC incidence in 4 Texas MSAs finds that prevention progress has stalled and that little to no progress on Black-White CRC inequality was achieved from 1999 to 2018. Texans have voted twice—first in 2007, and again in 2019—to establish and fund CPRIT, making cancer prevention a public priority. CPRIT recently identified ending cancer disparities as a priority [15]. Initiation of a new period of robust and widespread CRC prevention and closure of the Black-White gap warrant urgent attention from the *Texas Cancer Plan* as well as Texas cancer researchers. Ambitious and well-resourced CRC screening initiatives have succeeded elsewhere and may provide important lessons for Texas.

## Abbreviations

AAPC: average annual percent change
C5: Citywide Colon Cancer Control Coalition
CPRIT: Cancer Prevention and Research Institute of Texas
CRC: colorectal cancer
EC: excess case
JRM: Joinpoint regression modeling
MCMC: Markov chain Monte Carlo
MSA: metropolitan statistical area
PAR: proportion of attributable risk
RD: rate difference
RR: rate ratio
